# High Prevalence of Rifampicin Resistance Associated with Rural Residence and Very Low Bacillary Load among TB/HIV-Coinfected Patients at the National Tuberculosis Treatment Center in Uganda

**DOI:** 10.1155/2020/2508283

**Published:** 2020-07-25

**Authors:** Joseph Baruch Baluku, Pallen Mugabe, Rose Mulwana, Sylvia Nassozi, Richard Katuramu, William Worodria

**Affiliations:** ^1^Mulago National Referral Hospital, Pulmonology Division, P.O. Box 7051 Kampala, Uganda; ^2^Mildmay Uganda, P.O. Box 24985, Kampala, Uganda; ^3^Makerere University Lung Institute, P.O. Box 7749, Kampala, Uganda; ^4^National Tuberculosis and Leprosy Program, Ministry of Health, P.O. Box 7272 Kampala, Uganda

## Abstract

**Background:**

Rifampicin resistance (RR) is associated with mortality among tuberculosis (TB) patients coinfected with HIV. We compared the prevalence of RR among TB patients with and without HIV coinfection at the National Tuberculosis Treatment Center (NTTC) in Uganda, a TB/HIV high burdened country. We further determined associations of RR among TB/HIV-coinfected patients.

**Methods:**

In this secondary analysis, we included adult (≥18 years) bacteriologically confirmed TB patients that were enrolled in a cross-sectional study at the NTTC in Uganda between August 2017 and March 2018. TB, RR, and bacillary load were confirmed by the Xpert® MTB/RIF assay in the primary study. A very low bacillary load was defined as a cycle threshold value of >28. We compared the prevalence of RR among TB patients with and without HIV coinfection using Pearson's chi-square test. We performed logistic regression analysis to determine associations of RR among TB/HIV-coinfected patients.

**Results:**

Of the 303 patients, 182 (60.1%) were male, 111 (36.6%) had TB/HIV coinfection, and the median (interquartile range) age was 31 (25-39) years. RR was found among 58 (19.1%) patients. The prevalence of RR was 32.4% (36/111) (95% confidence interval (CI): 24-42) among TB/HIV-coinfected patients compared to 11.5% (22/192) (95% CI: 7–17) among HIV-negative TB patients (*p* < 0.001). Among TB/HIV-coinfected patients, those with RR were more likely to be rural residents (adjusted odds ratio (aOR): 5.24, 95% CI: 1.51–18.21, *p* = 0.009) and have a very low bacillary load (aOR: 13.52, 95% CI: 3.15–58.08, *p* < 0.001).

**Conclusion:**

There was a high prevalence of RR among TB/HIV-coinfected patients. RR was associated with rural residence and having a very low bacillary load among TB/HIV-coinfected patients. The findings highlight a need for universal access to drug susceptibility testing among TB/HIV-coinfected patients, especially in rural settings.

## 1. Background

Drug-resistant tuberculosis (DR-TB) is a growing public health concern, and over 500,000 cases of rifampicin-resistant tuberculosis were reported in 2018 globally [1]. In sub-Saharan Africa, the rate of decline in the burden of DR-TB is only 0.12% per year, yet the continent concurrently grapples with the HIV epidemic [2, 3]. The association between HIV and DR-TB is unclear partly due to the heterogeneity of studies [4]. One meta-analysis reported that HIV-infected patients are at a moderate risk of multidrug-resistant tuberculosis (MDR-TB)—resistance of *M. tuberculosis* to rifampicin and isoniazid [5]—while another meta-analysis performed among studies from sub-Saharan Africa showed no such association [6]. It is likely that the risk factors for DR-TB and HIV among tuberculosis (TB) patients are similar and the association of HIV with MDR-TB is a circumstantial convergence of the two epidemics in high-risk populations [7]. Nevertheless, MDR-TB is associated with a higher risk of mortality, treatment failure, and loss to follow-up among TB patients that are coinfected with HIV compared to HIV-negative patients [8, 9]. ART does not appear to improve DR-TB cure rates despite an increased ART uptake of 83% [8]. Moreover, HIV viral suppression among TB/HIV-coinfected patients with MDR-TB is reported to be 23%-64%, below the 90% global target [10, 11]. There is therefore a need for early detection of MDR-TB among TB/HIV-coinfected patients to enable early TB treatment initiation and avert the associated poor outcomes for both MDR-TB and HIV treatment. In Uganda, more than 41% of TB patients are coinfected with HIV [12]. Moreover, a DR-TB outbreak investigation in rural Uganda reported that 52% of MDR-TB patients compared to 32% of drug-sensitive TB patients had HIV coinfection, suggesting a higher risk of HIV infection among patients with MDR-TB (OR = 2.6, 95% CI: 1.1–6.1) [13].

Rifampicin is the most important antituberculosis agent [14]. Rifampicin resistance (RR) is a proxy of MDR-TB whereby 78% of TB patients with RR have MDR-TB [1]. Using RR as a proxy for MDR-TB reduces delays in treatment initiation that would occur if culture-based drug resistance testing is employed [15]. Moreover, RR as monoresistance is associated with mortality among TB/HIV-coinfected patients as well [16]. The prevalence and associations of RR among TB/HIV-coinfected TB patients in Uganda are not widely reported, yet the country is highly burdened with TB/HIV coinfection [1]. In this study, we compared the prevalence of RR among TB patients with and without HIV coinfection at the National Tuberculosis Treatment Center in Uganda. We further determined associations of RR among TB/HIV-coinfected patients.

## 2. Materials and Methods

### 2.1. Study Setting, Design, and Population

This was a secondary analysis of data of patients that were enrolled in a cross-sectional study [17] conducted at the National Tuberculosis Treatment Center (NTTC) in Uganda between August 2017 and March 2018. The primary study enrolled bacteriologically confirmed adult (>18 years) TB patients to determine the prevalence of malaria/TB coinfection. The NTTC is a center of excellence for drug-sensitive TB and DR-TB diagnosis and management at Mulago National Referral Hospital that is located in Kampala, the capital city of Uganda. Approximately 70% of TB patients initiating treatment at the NTTC are diagnosed at the same facility. The center also acts as a referral facility for complicated DR-TB cases from other 16 regional DR-TB care facilities in the country. The NTTC offers integrated TB/HIV services and conducts a weekly DR-TB/HIV clinic. In this secondary analysis, we included bacteriologically confirmed adult (≥18 years) TB patients with HIV and Xpert® MTB/RIF test results. We excluded patients for whom RR status was not reported or reported as indeterminate. In the primary study, participants were consecutively recruited at the NTTC.

### 2.2. Study Measurements

In the primary study, bacteriological confirmation of TB and RR was determined using an Xpert® MTB/RIF assay, a TB nucleic amplification test, on sputum samples. The Xpert® MTB/RIF assay grades bacillary load using cycle threshold (Ct) values as the following: very low (Ct > 28), low (Ct 22–28), medium (Ct 16–22), and high (Ct<16) [18]. HIV testing was performed on patients' serum using a rapid immunochromatographic rapid test (Alere Determine™ HIV-1/2) and confirmed by sequential testing with another immunochromatographic test (Chembio HIV 1/2 STAT-PAK™) according to the Uganda Ministry of Health HIV diagnostic algorithm [19]. In the primary study, socio-demographic characteristics and medical history were obtained through a face to face interview using a pretested questionnaire. A patient with 4 or more symptoms was arbitrarily assigned to have a high symptom burden. A full hemogram was performed on 5 ml of patient's whole blood using a hemoanalyser (Sysmex® automated hematology analyser XN series-XN 1000). The CD4 and CD8 T-cell counts were measured using a flow cytometer (BD FACSCalibur™) according to the manufacturer's instructions [20]. The reference ranges for the CD4/CD8 ratio used in this analysis are for adult Ugandans [21]. Other study methods are described elsewhere [17]. For this analysis, data were extracted from the primary study's dataset.

### 2.3. Statistical Analysis

The analysis was performed using Stata 15.1 (StataCorp, College Station, TX, USA). The prevalence of RR among TB/HIV-coinfected patients was determined as a proportion of TB/HIV-coinfected patients with RR to the total number of TB/HIV-coinfected patients. Similarly, the prevalence of RR among HIV-negative TB patients was determined as a proportion of HIV-negative TB patients with RR to the total number of TB patients without HIV coinfection. We compared the prevalence of RR among patients with or without HIV coinfection using Pearson's chi-square test.

Associations of RR among TB/HIV-coinfected patients were determined by logistic regression analysis. Variables that were found to have a *p* < 0.2 at bivariable logistic regression analysis were fitted into a logistic regression model that controlled for sex. Sex is noted to be a risk factor for RR and can modify the effect of other risk factors [4, 22]. In the multivariable logistic regression model, variables with a *p* < 0.05 at the 95% confidence interval were considered to be statistically significant and determined to be the associations of RR among TB/HIV-coinfected patients.

### 2.4. Ethical Approvals and Consent to Participate

Patients in the primary study provided written informed consent to the use of deidentified data for secondary analyses. The primary study was approved by the School of Medicine Research and Ethics Committee of Makerere University College of Health Sciences (REC REF 2017-087).

## 3. Results

### 3.1. Study Enrolment

**Figure 1:**
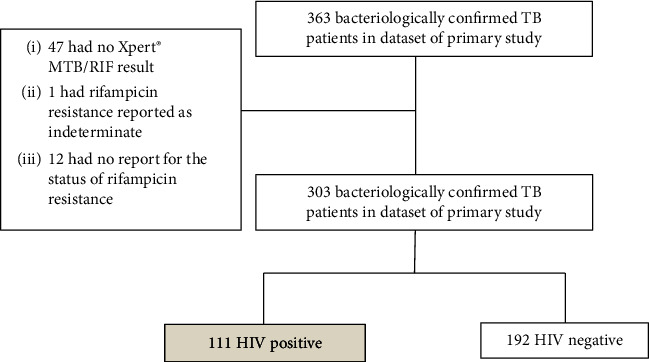
Study flow diagram.

Of the 363 patients in the dataset, we included 303 patients that met the study's inclusion criteria. Of the 60 participants that were excluded, 19 (31.7%) were HIV-infected while 14 (23.3%) were rural residents. The study flow diagram is shown in Figure 1.

### 3.2. Characteristics of Study Patients

Of the 303 patients, 182 (60.1%) were males and median (interquartile range) age was 31 (25-39) years. RR was found among 58 (19.1%) patients while TB/HIV coinfection was found among 111 (36.6%) patients. There was no difference in the HIV status (*p* = 0.463) and residence (*p* = 0.100) of participants that were excluded and those included in this analysis. The characteristics of TB/HIV-coinfected patients are shown in Table 1.

### 3.3. Comparison of the Prevalence of RR among TB/HIV-Coinfected and TB-Monoinfected Patients

The prevalence of RR was 32.4% (36/111) (95% CI: 24-42) among TB/HIV-coinfected patients compared to 11.5% (22/192) (95% CI: 7–17) among TB patients without HIV coinfection (*p* < 0.001).

### 3.4. Associations of RR among TB/HIV-Coinfected Patients

Among TB/HIV-coinfected patients, there was a statistically significant association of RR with rural residence (adjusted odds ratio (aOR): 5.24, 95% confidence interval (CI): 1.51–18.21, *p* = 0.009) and having a very low bacillary load (aOR: 13.52, 95% CI: 3.15–58.08, *p* < 0.001). There was no statistically significant association with other variables as shown in Table 2.

## 4. Discussion

In this study, we compared the prevalence of RR among TB patients with or without HIV coinfection at the NTTC in Uganda and determined associations of RR among TB/HIV-coinfected patients. We found the prevalence of RR among TB/HIV-coinfected patients to be three times higher than among TB patients without HIV coinfection (32.4% vs. 11.5%, *p* < 0.001). We also found that TB/HIV-coinfected patients with RR were more likely to be rural residents and have a very low bacillary load.

The high prevalence of RR among TB/HIV-coinfected patients is concerning because it is associated with poor TB treatment outcomes [8]. This highlights the need for universal access to drug susceptibility testing (DST) among TB/HIV-coinfected patients in resource-limited settings where only 35% of new TB cases have a rifampicin DST performed [23]. Further, TB patients with RR from rural areas are 2–3 times more likely to experience delays in treatment initiation and a high loss to follow-up compared to urban dwellers [24–26]. This could increase the risk of community spread of DR-TB. Universal testing with Xpert® MTB/RIF would otherwise reduce diagnosis delays in rural settings if implemented with fidelity [27]. In Uganda, the linking of rural health centers to an Xpert® MTB/RIF testing hub using sample transporters seems to be ineffective since only 1.8% of presumptive TB cases receive Xpert® MTB/RIF testing as a first-line TB diagnostic test [28]. The association of rural residence with DR-TB is equivocal with some studies reporting urban residence to be a risk factor for DR-TB [4]. Interestingly, a higher proportion of DR-TB patients was also reported to hail from rural areas in Netherlands, a high income country with a low TB prevalence [29]. The association of RR with having very low bacillary load among TB/HIV-coinfected patients may further compound the challenge of bacteriological confirmation of TB among HIV-infected patients with presumptive DR-TB [18, 30]. There is therefore a need for more sensitive TB diagnostic tests among HIV-infected patients with presumptive DR-TB. There is scarcity of literature reporting the relationship between mycobacterial loads and RR among TB/HIV coinfected [31]. Among HIV-negative TB patients, high bacillary loads are associated with drug-resistant TB [32]. Lower bacillary loads among TB/HIV coinfected with RR (when compared to TB/HIV coinfected without RR) could be due to low biological fitness of *Mycobacterium tuberculosis* strains with RR, whereby these strains have lower growth rates [33]. However, this needs to be further studied considering that TB/HIV-coinfected patients with DR-TB do not seem to have more resistance-conferring mutations with fitness cost when compared to HIV-negative counterparts [34].

Similar to our findings, Sethi et al. found the prevalence of RR to be higher among TB/HIV-coinfected patients (27.3%) than TB patients without HIV coinfection (15.4%) in India [35]. Our findings are also in agreement with Timire et al., who found the prevalence of RR to be 7.3% vs. 2.8% among TB/HIV-coinfected and HIV-negative TB patients in Zimbabwe, respectively [36]. Denue et al. reported similar findings (7.0% vs. 4.8%) in Nigeria [37]. However, the prevalence of 32.4% of RR among TB/HIV-coinfected patients in our study is higher than in the aforementioned studies. Our study was conducted at tertiary referral facility. This might have overestimated the prevalence of RR owing to referral bias of TB/HIV-coinfected patients who are likely to have complications that require referral [38]. Interestingly, the majority (83%) of the TB/HIV-coinfected patients with RR had no history of TB treatment. The high prevalence of primary DR-TB among TB/HIV-coinfected patients can be explained by the rapid progression of TB infection to TB disease among HIV-positive individuals in the context of a DR-TB outbreak that has been reported to occur in rural Uganda [13]. It is plausible that diagnosis and treatment delays coupled with fragile health systems in rural areas fuel community transmission of DR-TB to vulnerable groups such as HIV-infected individuals [13].

In contrast to our study findings, Ukwamedua et al. found the prevalence of RR to be higher among TB patients who are HIV seronegative than TB/HIV-coinfected patients (7.8% vs. 5%) in Nigeria [39]. However, their sample included paediatric patients, and for more than 25% of participants with RR, the HIV status was unknown. Arega et al. found no difference in the prevalence of RR between the TB patients with or without HIV coinfection in Ethiopia although the HIV status was unknown for 90% of their study population and they included patients < 15 years of age as well [40]. Further, the difference in the prevalence was not statistically significant in these studies.

A key limitation of our study is the small sample size that limits the precision of estimating the effect size of the associations. Moreover, some risk factors for DR-TB such as history of incarceration were not evaluated due to missing data. We therefore recommend an evaluation of the associations of RR among TB/HIV-coinfected patients with a larger sample size.

## 5. Conclusion

There is a high prevalence of RR among TB/HIV-coinfected patients. RR among TB/HIV-coinfected patients is associated with a very low bacillary load and rural residence. This highlights a need for universal drug susceptibility testing among TB/HIV-coinfected patients, especially in rural settings.

## Figures and Tables

**Table 1:** Characteristics of TB/HIV-coinfected patients.

Characteristic	Rifampicin resistance (RR) not detected (*n* = 75) (%)	RR detected (*n* = 36) (%)	Total (*N* = 111) (%)	*p* value^ɣ^
Sex				
Male	40 (53.3)	16 (44.4)	56 (50.5)	0.381
Female	35 (46.7)	20 (55.6)	55 (49.5)	
Age (median (IQR))	33 (27-39)	34.5 (27-40)	31 (25–39)	0.420^‡^
Residence				
Urban	57 (76.0)	17 (47.2)	74 (66.7)	0.003
Rural	18 (24.0)	19 (52.8)	37 (33.3)	
Antiretroviral therapy (ART)				
No current ART use	45 (60.0)	15 (41.7)	60 (54.1)	0.070
Current ART use	30 (40.0)	21 (58.3)	51 (45.9)	
Current cotrimoxazole (CTX) use				
Yes	47 (62.7)	29 (80.6)	76 (68.5)	0.058
No	28 (37.3)	7 (19.4)	35 (31.5)	
CTX and ART use combined				
Yes	24 (32.0)	19 (52.8)	43 (38.7)	0.035
No	51 (68.0)	17 (47.2)	68 (61.3)	
History of TB treatment				
Yes	12 (16.0)	6 (16.7)	18 (16.2)	1.000
No	63 (84.0)	30 (83.3)	93 (83.8)	
Cough				
Yes	75 (100.0)	31 (86.1)	106 (95.5)	0.003
No	0 (0.0)	5 (13.9)	5 (4.5)	
Night sweats				
Yes	55 (73.3)	22 (61.1)	77 (69.4)	0.191
No	20 (26.7)	14 (38.9)	34 (30.6)	
Weight loss				
Yes	60 (80.0)	20 (55.6)	80 (72.1)	0.007
No	15 (20.0)	16 (44.4)	31 (27.9)	
TB symptom burden				
≥4 symptoms	43 (57.3)	15 (41.7)	58 (52.2)	0.122
<4 symptoms	32 (42.7)	21 (58.3)	53 (47.8)	
Bacillary load				
Very low	8 (11.0)	16 (48.5)	24 (22.6)	<0.001
Low/medium/high	65 (89.0)	17 (51.5)	82 (77.4)	
Temperature				
Hypothermia	21 (28.4)	14 (38.9)	35 (31.8)	0.038
Normal	38 (51.4)	21 (58.3)	59 (53.6)	
Hyperthermia	15 (20.2)	1 (2.8)	16 (14.6)	
CD4 (median (IQR))	182 (68-325)	280 (119.5-523.5)	433 (220-718)	0.138^‡^
CD8 (median (IQR))	466 (254-751)	542.5 (333-911.5)	414 (253-629)	0.420^‡^
CD4/CD8 ratio^†^				
<0.52	58 (77.3)	22 (61.1)	80 (72.1)	0.075
0.52-4.1	17 (22.7)	14 (38.9)	31 (27.9)	
WBC count*^ß^* (median (IQR))	5.89 (4.03-8.61)	4.89 (4.08-6.61)	6.73 (4.80-9.35)	0.115^‡^
Hemoglobin^§^ (median (IQR))	10.7 (9.1-12.5)	11.5 (9.1-13.5)	12.2 (10.2-13.9)	0.195^‡^
MCV^*ψ*^ (median (IQR))	78.4 (71.2-87.6)	88.0 (77.1-96.9)	78.7 (71.5-87.9)	0.133^‡^

^ɣ^ *p* value is derived from Pearson's chi-square test; ^‡^*p* value from nonparametric median test. TB: tuberculosis; WBC: white blood cell count; MCV: mean corpuscular volume; CD: cluster of differentiation; IQR: interquartile range. ^†^Cutoff ranges are according to Ugandan population estimate [21]; *^ß^*×10^3^ cells per microliter; ^§^grams per deciliter; ^*ψ*^femtoliters.

**Table 2:** Multivariable logistic regression model for associations of RR among TB/HIV-coinfected patients.

Characteristic	Crude odds ratio 95% confidence interval	*p* value	Adjusted odds ratio 95% confidence interval	*p* value
Sex^†^				
Male	1		1	
Female	1.43 (0.64-3.18)	0.381	1.53 (0.45–5.26)	0.498
Residence				
Urban	1		1	
Rural	3.54 (1.52–8.21)	0.003	5.24 (1.51–18.21)	0.009
CTX and ART use				
No	1		1	
Yes	2.38 (1.05-5.36)	0.037	2.57 (0.78–8.47)	0.120
Night sweats				
No	1		1	
Yes	0.57 (0.25-1.33)	0.193	1.08 (0.24–4.92)	0.921
Weight loss				
No	1		1	
Yes	0.31 (0.13-0.74)	0.009	0.26 (0.07-1.01)	0.052
TB symptom burden				
<4	1		1	
≥4	0.53 (0.24-1.19)	0.124	1.20 (0.30-4.76)	0.797
Bacillary load				
Low/medium/high	1		1	
Very low	7.65 (2.81–20.84)	<0.001	13.52 (3.15–58.08)	<0.001
Temperature				
Hypothermia	1		1	
Normal	0.83 (0.35-1.96)	0.669	0.29 (0.07-1.17)	0.082
Hyperthermic	0.10 (0.01-0.85)	0.034	0.14 (0.01-2.19)	0.160
CD4/CD8 ratio				
<0.52	1		1	
0.52-4.1	2.17 (0.92-5.14)	0.078	1.27 (0.33–4.93)	0.732
White blood cell count	0.91 (0.80-1.04)	0.186	1.05 (0.86-1.29)	0.611
Mean corpuscular volume	1.05 (1.01-1.09)	0.011	1.06 (0.99-1.13)	0.085

TB: tuberculosis; CD: cluster of differentiation; CTX: cotrimoxazole; ART: antiretroviral therapy. ^†^Included in the model due to effect on other risk factors for rifampicin-resistant TB [4, 22]. Note: Nagelkerke *R*^2^ = 0.527, that is, the covariates in the model explain 53% variation in RR among HIV/TB-coinfected patients. Cox Snell *R*^2^ = 0.374, that is, the covariates explain 37% of variation when using this criteria.

## Data Availability

The data used to support the findings of this study are available from the corresponding author upon request.

## References

[1] World Health Organization (2019). *Global tuberculosis report 2019*.

[2] Musa B. M., Adamu A. L., Galadanci N. A., Zubayr B., Odoh C. N., Aliyu M. H. (2017). Trends in prevalence of multi drug resistant tuberculosis in sub-Saharan Africa: a systematic review and meta-analysis. *PLoS One*.

[3] Kharsany A. B. M., Karim Q. A. (2016). HIV Infection and AIDS in Sub-Saharan Africa: Current Status, Challenges and Opportunities. *The Open AIDS Journal*.

[4] Pradipta I. S., Forsman L. D., Bruchfeld J., Hak E., Alffenaar J.-W. (2018). Risk factors of multidrug-resistant tuberculosis: a global systematic review and meta-analysis. *Journal of Infection*.

[5] Mesfin Y. M., Hailemariam D., Biadglign S., Kibret K. T. (2014). Association between HIV/AIDS and multi-drug resistance tuberculosis: a systematic review and meta-analysis. *PLoS ONE*.

[6] Berhan A., Berhan Y., Yizengaw D. (2013). A meta-analysis of drug resistant tuberculosis in sub-Saharan Africa: how strongly associated with previous treatment and HIV co-infection?. *Ethiopian Journal of Health Sciences*.

[7] Hof S., Tursynbayeva A., Abildaev T., Adenov M., Pak S., Ismailov S. (2015). HIV and multidrug-resistant tuberculosis: overlapping risk factors. *European Respiratory Journal*.

[8] Chem E. D., Van Hout M. C., Hope V. (2019). Treatment outcomes and antiretroviral uptake in multidrug-resistant tuberculosis and HIV co-infected patients in sub Saharan Africa: a systematic review and meta-analysis. *BMC Infectious Diseases*.

[9] Samuels J. P., Sood A., Campbell J. R., Khan F. A., Johnston J. C. (2018). Comorbidities and treatment outcomes in multidrug resistant tuberculosis: a systematic review and meta-analysis. *Scientific Reports*.

[10] Brust J. C. M., Shah N. S., Mlisana K. (2018). Improved survival and cure rates with concurrent treatment for multidrug-resistant tuberculosis–human immunodeficiency virus coinfection in South Africa. *Clinical Infectious Diseases*.

[11] Efsen A. M. W., Schultze A., Miller R. F. (2018). Management of MDR-TB in HIV co-infected patients in Eastern Europe: results from the TB:HIV study. *The Journal of Infection*.

[12] Kirenga B. J., Ssengooba W., Muwonge C. (2015). Tuberculosis risk factors among tuberculosis patients in Kampala, Uganda: implications for tuberculosis control. *BMC Public Health*.

[13] Okethwangu D., Birungi D., Biribawa C. (2019). Multidrug-resistant tuberculosis outbreak associated with poor treatment adherence and delayed treatment: Arua District, Uganda, 2013–2017. *BMC Infectious Diseases*.

[14] Arbex M. A., de Castro Lima Varella M., de Siqueira H. R., de Mello F. A. F. (2010). Drogas antituberculose: interações medicamentosas, efeitos adversos e utilização em situações especiais - parte 1: fármacos de primeira linha. *Jornal Brasileiro de Pneumologia*.

[15] Santos A. P., Leung J., Malaquias T., Vieira M. A. M. D. S., Kritski A., Mello F. C. Q. (2020). The reliability of rifampicin resistance identified on Xpert? MTB/RIF as a proxy for multidrug-resistant tuberculosis (MDR-TB) in a reference center for MDR-TB in Rio de Janeiro, Brazil. In: C62 TUBERCULOSIS: BENCH TO BEDSIDE. *American Thoracic Society*.

[16] Sharling L., Marks S. M., Goodman M., Chorba T., Mase S. (2020). Rifampin-resistant tuberculosis in the United States, 1998–2014. *Clinical Infectious Diseases*.

[17] Baluku J. B., Nassozi S., Gyagenda B. (2019). Prevalence of malaria and TB coinfection at a national tuberculosis treatment centre in Uganda. *Journal of Tropical Medicine*.

[18] Lawn S. D., Nicol M. P. (2011). Xpert® MTB/RIF assay: development, evaluation and implementation of a new rapid molecular diagnostic for tuberculosis and rifampicin resistance. *Future Microbiology*.

[19] Ministry of Health (2016). *National HIV testing services policy and implementation guidelines*.

[20] Biosciences B. (2016). *BD Multitest IMK Kit*.

[21] Nanzigu S., Waako P., Petzold M. (2011). CD4-T-lymphocyte reference ranges in Uganda and its influencing factors. *Laboratory Medicine*.

[22] Faustini A., Hall A. J., Perucci C. A. (2006). Risk factors for multidrug resistant tuberculosis in Europe: a systematic review. *Thorax*.

[23] Ismail N., Ismail F., Omar S. V. (2018). Drug resistant tuberculosis in Africa: current status, gaps and opportunities. *African Journal of Laboratory Medicine*.

[24] Soeroto A. Y., Lestari B. W., Santoso P. (2019). Evaluation of Xpert *MTB-RIF* guided diagnosis and treatment of rifampicin-resistant tuberculosis in Indonesia: a retrospective cohort study. *PLoS One*.

[25] Javaid A., Shaheen Z., Shafqat M., Khan A. H., Ahmad N. (2017). Risk factors for high death and loss-to-follow-up rates among patients with multidrug-resistant tuberculosis at a programmatic management unit. *American Journal of Infection Control*.

[26] Molie T., Teklemariam Z., Klinkenberg E. (2019). Intensive phase treatment outcome and associated factors among patients treated for multi drug resistant tuberculosis in Ethiopia: a retrospective cohort study. *BMC Infectious Diseases*.

[27] Iruedo J., O’Mahony D., Mabunda S., Wright G., Cawe B. (2017). The effect of the Xpert MTB/RIF test on the time to MDR-TB treatment initiation in a rural setting: a cohort study in South Africa’s Eastern Cape Province. *BMC Infectious Diseases*.

[28] Nalugwa T., Shete P. B., Nantale M. (2020). Challenges with scale-up of GeneXpert MTB/RIF® in Uganda: a health systems perspective. *BMC Health Services Research*.

[29] Pradipta I. S., van’t Boveneind-Vrubleuskaya N., Akkerman O. W., Alffenaar J.-W. C., Hak E. (2019). Treatment outcomes of drug-resistant tuberculosis in the Netherlands, 2005–2015. *Antimicrobial Resistance & Infection Control*.

[30] Hanrahan C. F., Theron G., Bassett J. (2014). Xpert MTB/RIF as a measure of sputum bacillary burden. Variation by HIV status and immunosuppression. *American Journal of Respiratory and Critical Care Medicine*.

[31] Khan P. Y., Yates T. A., Osman M. (2019). Transmission of drug-resistant tuberculosis in HIV-endemic settings. *The Lancet Infectious Diseases*.

[32] Sander M. S., Vuchas C. Y., Numfor H. N. (2016). Sputum bacterial load predicts multidrug-resistant tuberculosis in retreatment patients: a case-control study. *The International Journal of Tuberculosis and Lung Disease*.

[33] Zhan L., Wang J., Wang L., Qin C. (2020). The correlation of drug resistance and virulence in *Mycobacterium tuberculosis*. *Biosafety and Health*.

[34] Ssengooba W., Lukoye D., Meehan C. J. (2017). Tuberculosis resistance-conferring mutations with fitness cost among HIV-positive individuals in Uganda. *The International Journal of Tuberculosis and Lung Disease*.

[35] Sethi S., Mewara A., Dhatwalia S. K. (2013). Prevalence of multidrug resistance in *Mycobacterium tuberculosis* isolates from HIV seropositive and seronegative patients with pulmonary tuberculosis in north India. *BMC Infectious Diseases*.

[36] Timire C., Metcalfe J. Z., Chirenda J. (2019). Prevalence of drug-resistant tuberculosis in Zimbabwe: a health facility-based cross-sectional survey. *International Journal of Infectious Diseases*.

[37] Denue B. A., Miyanacha W. J., Wudiri Z., Alkali M. B., Goni B. W., Akawu C. B. (2018). Molecular detection of sputum *Mycobacterium tuberculosis*/rifampicin resistance among presumptive pulmonary tuberculosis cases in Borno state, North-Eastern Nigeria. *Port Harcourt Medical Journal*.

[38] Esmail A., Sabur N. F., Okpechi I., Dheda K. (2018). Management of drug-resistant tuberculosis in special sub-populations including those with HIV co-infection, pregnancy, diabetes, organ-specific dysfunction, and in the critically ill. *Journal of Thoracic Disease*.

[39] Ukwamedua H., Omote V., Etaghene J., Oseji M. E., Agwai I. C., Agbroko H. (2019). Rifampicin resistance among notified pulmonary tuberculosis (PTB) cases in south-southern Nigeria. *Heliyon*.

[40] Arega B., Menbere F., Getachew Y. (2019). Prevalence of rifampicin resistant *Mycobacterium tuberculosis* among presumptive tuberculosis patients in selected governmental hospitals in Addis Ababa, Ethiopia. *BMC Infectious Diseases*.

